# The Association of Integrins β3, β4, and αVβ5 on Exosomes, CTCs and Tumor Cells with Localization of Distant Metastasis in Breast Cancer Patients

**DOI:** 10.3390/ijms24032929

**Published:** 2023-02-02

**Authors:** Evgeniya S. Grigoryeva, Luibov A. Tashireva, Olga E. Savelieva, Marina V. Zavyalova, Nataliya O. Popova, Gleb A. Kuznetsov, Elena S. Andryuhova, Vladimir M. Perelmuter

**Affiliations:** 1 The Laboratory of Molecular Therapy of Cancer, Cancer Research Institute, Tomsk National Research Medical Center, Russian Academy of Sciences, 634009 Tomsk, Russia; 2 The Laboratory of Molecular Oncology and Immunology, Cancer Research Institute, Tomsk National Research Medical Center, Russian Academy of Sciences, 634009 Tomsk, Russia; 3 The Department of General and Molecular Pathology, Cancer Research Institute, Tomsk National Research Medical Center, Russian Academy of Sciences, 634009 Tomsk, Russia; 4 Research Center, Saint-Petersburg State Pediatric Medical University, 194100 Saint-Petersburg, Russia; 5 The Department of Chemotherapy, Cancer Research Institute, Tomsk National Research Medical Center, Russian Academy of Sciences, 634009 Tomsk, Russia

**Keywords:** breast cancer, metastasis, circulating tumor cells, exosomes, integrin

## Abstract

Integrins are cell adhesion receptors, which play a role in breast cancer invasion, angiogenesis, and metastasis. Moreover, it has been shown that exosomal integrins provide organotropic metastasis in a mouse model. In our study, we aimed to investigate the expression of integrins β3, β4, and αVβ5 on exosomes and tumor cells (circulating tumor cells and primary tumor) and their association with the localization of distant metastasis. We confirmed the association of exosomal integrin β4 with lung metastasis in breast cancer patients. However, we were unable to evaluate the role of integrin β3 in brain metastasis due to the rarity of this localization. We established no association of exosomal integrin αVβ5 with liver metastasis in our cohort of breast cancer patients. The further evaluation of β3, β4, and αVβ5 integrin expression on CTCs revealed an association of integrin β4 and αVβ5 with liver, but not the lung metastases. Integrin β4 in the primary tumor was associated with liver metastasis. Furthermore, an in-depth analysis of phenotypic characteristics of β4+ tumor cells revealed a significantly increased proportion of E-cadherin+ and CD44+CD24- cells in patients with liver metastases compared to patients with lung or no distant metastases.

## 1. Introduction

The development of distant metastases in most cases becomes crucial for patients with breast cancer. Sufficient data have been accumulated regarding the localization of breast cancer metastases. Breast cancer metastases frequently occur in bones, lungs, liver, and less often in the brain while other localizations are extremely rare. The organotropism of breast cancer metastases is still an unresolved problem. There is evidence, mostly experimental, about the role of integrins in determining the localization of metastases.

Integrins are heterodimeric transmembrane glycoproteins, with the α and β subunits having extracellular, transmembrane, and cytoplasmic domains. While the extracellular domain serves as a receptor for adhesion proteins and growth factors in the extracellular matrix (ECM), the cytoplasmic domain is associated with the cytoskeleton and cellular signaling pathways that are involved in invasion and angiogenesis [1]. It is important to emphasize that each of these integrin functions contributes to the metastatic potential of tumor cells.

Hoshino et al. (2015) conducted an extremely elegant experiment in vivo, which convincingly demonstrated that the exosomal integrins could play a crucial role in the organotropism of distant metastasis in a breast cancer model. By using an animal model, it has been shown that exosomal integrin β3 targets tumor cells to the brain, β4—to the lungs, and αVβ5—to the liver [2]. Specific exosomal integrins selectively accumulated in the ECM in the lungs and liver. Specifically, integrin α6β4 in S100A4-positive lung stromal cells enriched by laminin, integrin αvβ5 in F4/80+ liver stromal macrophages, which contain fibronectin. It is well-known that exosomes can act as a transporter of various bioactive molecules. Therefore, it has been shown that exosomes can carry phlogogenic agents, such as pro-caspase-1, IL-1, 6, 18, and components of inflammasomes [3]. Thuswise, exosomes of tumor cells contain molecules that in distant organs contribute to increased vascular permeability, to the development of inflammation, and to the recruitment of bone marrow progenitor cells. All these mechanisms are involved in the formation of premetastatic niches in a distant organ.

Despite the available data, the majority of the mechanisms underlying the role of exosomes with specific integrins in organotropic metastasis remain unexplored. The question arises whether it is possible that the integrin-mediated targeting mechanism is universal, given the fact that exosomes are produced by tumor cells (at the primary site or in circulation). Circulating tumor cells (CTCs) are the main drivers of cancer recurrence and metastasis. However, there is practically no data on the expression of integrins on CTCs. 

The expression of integrins in tumor cells is well studied, but this is mainly related to cell cultures, and not to the primary tumor. Therefore, it is known that expression of β3 in breast carcinoma cells promotes both lymph nodes [4] and bone metastases [5,6]. Moreover, αvβ3 may participate in enhancing tumor cell adherence to the pulmonary vasculature in 66cl4 mammary carcinoma line [5]. In the MDA-MB-435 human cancer cells model integrin αvβ3 critically enhances tumor cell growth in the brain, although it is not required for growth in the mammary fat pads [7]. The integrin αvβ5 enhanced cell migration and liver metastasis of colon carcinomas [8]. Upregulation of integrin β4 is associated with enhanced tumor growth and metastasis to the lung in the hepatocellular carcinoma cell line [9]. In the melanoma model, αvβ3 integrin targets the circulating melanoma cells to the lungs [10]. Thus, data on the expression of integrins are rather scattered and there is no understanding of how the expression of the same integrins correlates in primary tumor cells, in CTCs, and in exosomes.

Moreover, other questions arise. Can several different integrins be expressed on the same exosome? What factor determines the presence of metastases in one organ and the absence in others, if there are different integrins expressed on the exosomes and tumor cells? The goal of the current study was to investigate these issues in breast cancer patients.

## 2. Results

We analyzed two cohorts of breast cancer patients: the first cohort included 18 patients with M1 status with a predominance of luminal B HER2- (in 38.8%), T4 (44.4%) patients, with lymph node involvement in 72% of patients (Table 1). The second cohort included 48 patients with M0 status with a predominance of luminal B HER2+ (in 41.6%), T2 (58.3%), with lymph node involvement in 42% of patients. In the second group, 12 patients developed metastases during the follow-up period. Full clinicopathological data are represented in Table 1. 

### 2.1. Exosomal Expression of β3, β4, and αVβ5 Integrins in Breast Cancer Patients 

**Figure 1:**
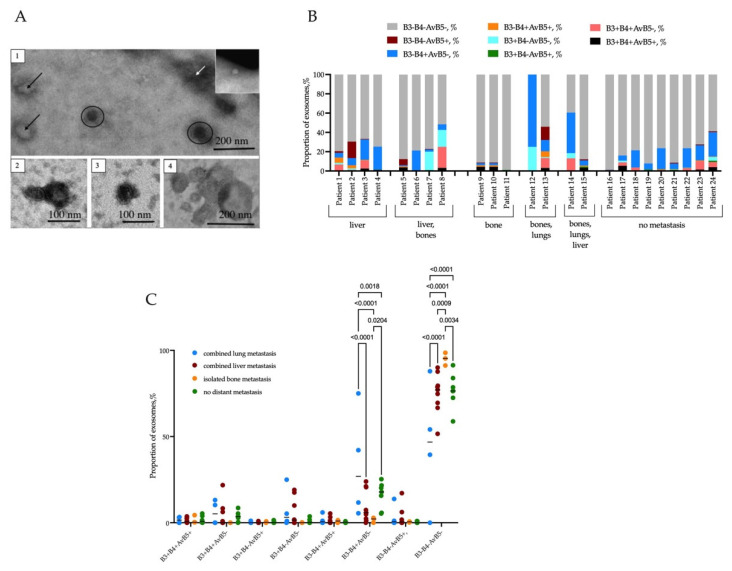
(**A**) A representative image of microvesicles isolated from the plasma of a breast cancer patient: ovals show exosomes, white arrows show protein aggregates, black arrows show non-vesicles (40–80 nm). The inset in the right angle shows a non-vesicle (20–40 nm) and 2, 3 represent exosomes, while 4 represents large vesicles (100–200 nm). (**B**) Intrapersonal variants of exosomal integrins co-expression in each breast cancer patient. (**C**) Proportions of exosomes co-expressed β3, β4, and αVβ5 integrins in breast cancer patients with metastasis in different organs. Samples were tested for statistical outliers, then one-way ANOVA was used to compare the means of independent groups.

Exosome isolation has been confirmed by TEM (Figure 1A). Frequency of occurrence of β3, β4, and αVβ5 integrins co-expression in breast cancer patients represented in Table 2. The most common variants were exosomes without integrin expression and with mono-expression of integrin β4, which occurs in almost all cases. Mono-expression of αVβ5 and β3 integrins occurred only in half of the cases. Co-expression of two and three integrins was detected in the range from 37.5 to 79.2% of the studied cases.

We evaluated the co-expression of β3, β4, and αVβ5 integrins on the exosomes of each patient (Figure 1B). It turned out that β3+β4+αVβ5+ and β3-β4+αVβ5- subpopulations of exosomes were represented in all patients with liver-only metastasis. While β3+β4-αVβ5+ exosomes were absent in three out of four patients with liver metastasis. The rest of the phenotypic variants occurred in at least two patients. 

In patients with liver and bone metastases β3-β4+αVβ5- exosomes were detected in all cases, while β3+β4+αVβ5+ exosomes were detected in three out of four cases. Exosomes with β3-β4+αVβ5+, β3+β4-αVβ5+ and β3+β4+αVβ5- phenotypes occurred only once (Patients 5, 7, 8, respectively). The rest of phenotypic variants occurred in at least two patients. 

Patients with isolated bone metastases were characterized by the smallest proportion of exosomes with β3, β4, and αVβ5 integrins. In all patients, we observed exosomes with β3+β4+αVβ5+ and β3-β4-αVβ5+ phenotypes. At the same time β3+β4+αVβ5- exosomes were absent in all cases. Exosomes with β3+β4-αVβ5+ and β3+β4-αVβ5- phenotypes were detected only in patient 11, which has no β3-β4+αVβ5+ and β3-β4+αVβ5- exosomes. In Patients 9 and 10 the pattern was reversed. Specifically, phenotypes β3+β4-αVβ5+ and β3+β4-αVβ5- were absent, while β3-β4+αVβ5+ and β3-β4+αVβ5- phenotypes were observed in two out of three patients with bone metastasis.

Groups of patients with combined bone/lung and liver/lung/bone metastases were characterized by a significant proportion of β3-β4+αVβ5- exosomes. 

In non-metastatic patients, the most prevalent integrin phenotype was β3-β4+αVβ5- and was detected in all patients. While the rarest phenotypes β3-β4+αVβ5+ and β3-β4-αVβ5+ occurred in four out of nine cases.

Therefore, exosomes of 13 out of 15 metastatic patients simultaneously expressed all three types of integrins. Exosomes detected in non-metastatic patients also expressed β3, β4, and αVβ5 integrins in all cases, excluding one patient. The diversity of exosomal integrins co-expression varied from two to seven phenotypes out of eight possible in metastatic patients. Patients without metastasis in the follow-up period revealed a similar pattern: the diversity of phenotypes varied from three to eight phenotypes. 

We grouped metastatic patients as follows: a group of breast cancer patients with combined lung metastasis (including patients with other localization of metastasis); a group of patients with combined liver metastasis (including patients with other localization of metastasis, excluding metastases in lung), and a group of patients without metastases in the 1-year follow up period. Frequency of occurrence and proportion of exosomal co-expression of β3, β4, and αVβ5 integrins in metastatic and non-metastatic breast cancer patients represented in Figure 1C. 

**Figure 2:**
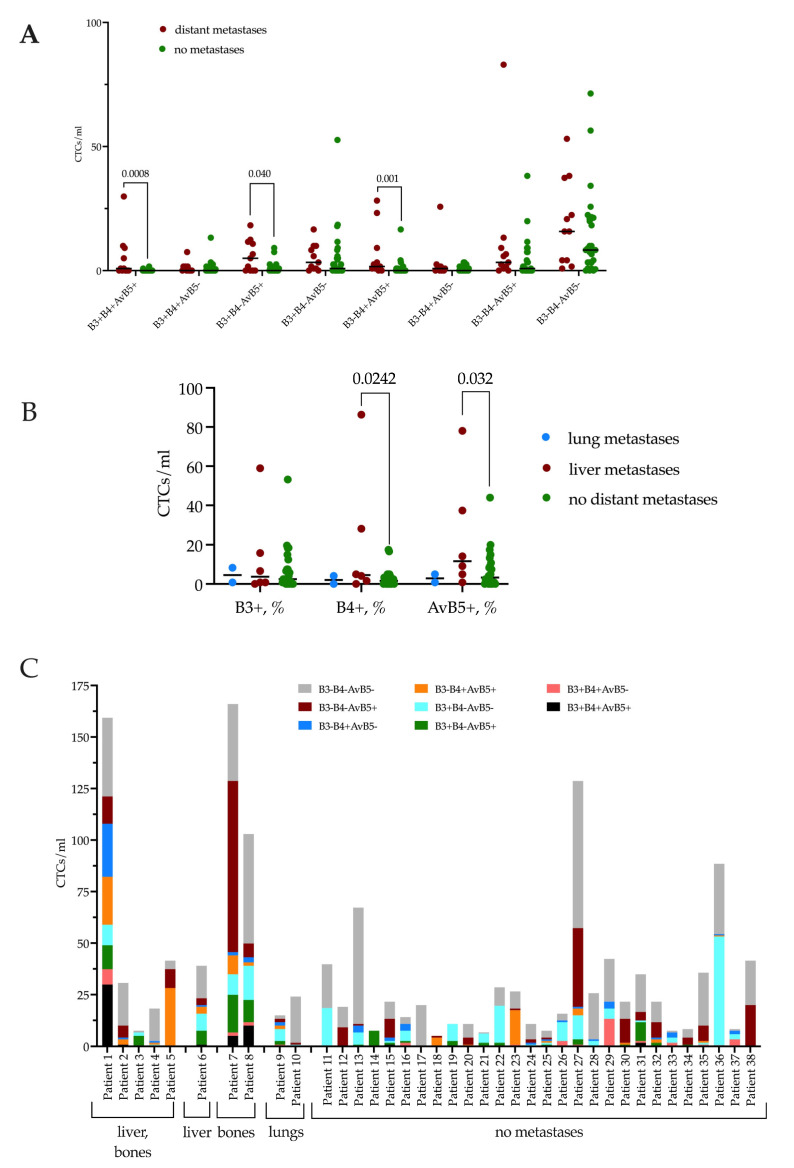
(**A**) Number of CTC co-expressed β3, β4, and αVβ5 integrins in breast cancer patients. (**B**) Number of CTC co-expressed β3, β4, and αVβ5 integrins in breast cancer patients with distant metastases in different organs. (**C**) Intrapersonal variants of integrins co-expression in CTCs of each breast cancer patient. Samples were tested for statistical outliers, then one-way ANOVA was used to compare the means of independent groups.

Patients with combined lung metastases had a higher proportion of exosomes with a β3-β4+αVβ5- phenotype compared to patients without metastases and patients with combined liver metastases and isolated bone metastases (*p* = 0.0018, *p* < 0.0001, *p* < 0.0001, respectively). On the contrary, the proportion of exosomes without expression of integrins (β3-β4-αVβ5-) was lower in patients with combined lung metastases in relation to the same groups (*p* < 0.0001, *p* < 0.0001, *p* < 0.0001). In patients with isolated bone metastases, the changes were the opposite. Namely, the proportion of β3-β4+αVβ5- exosomes in patients with isolated bone metastases was minimal: lower than in patients without metastases (*p* = 0.0204). While the proportion of exosomes without integrin expression (β3-β4-αVβ5-) was higher compared to patients without metastases (*p* = 0.0034), as well as compared to the group with combined liver metastases (*p* = 0.0009) and combined lung metastases (*p* = 0.0001) (Figure 2, panel B).

### 2.2. Frequency and Level of CTC with Expression of β3, β4 and αVβ5 Integrins in Breast Cancer Patients

The frequency of occurrence of β3, β4, and αVβ5 integrins co-expression in the CTCs of breast cancer patients is represented in Table 3. As in the case of exosome analysis, CTCs without integrin expression were detected more often. Compared to exosomes, CTCs with co-expression of all three integrins were significantly less common (18.4% (7/38) and 79.2% (19/24), respectively, *p* < 0.0001). Likewise, mono-expression of β4 on CTCs was less common than on exosomes (52.6% (20/38) and 95.8% (23/24), respectively, *p* = 0.0002). The frequencies of β3 and αVβ5 mono-expression and co-expression variants β3+β4+αvβ5-, β3+β4-αvβ5+, and β3-β4+αvβ5+ did not differ significantly on CTCs and exosomes.

Comparative analysis revealed an increase in the number of β3+β4+αVβ5+, β3+β4-αVβ5+, β3-β4+αVβ5+ CTCs (*p* = 0.008, *p* = 0.04, *p* = 0.001, respectively) in the group of patients with distant metastases. The median of β3+β4+αVβ5+ CTCs in patients with distant metastases was 0.83 (0; 9.130), β3+β4-αVβ5+ CTCs—4.98 (0; 11.620), β3-β4+αVβ5+ CTCs—1.66 (0.83; 9130) cells per ml of peripheral blood, while patients without metastases had 0 (0; 0), 0 (0; 1.453), and 0 (0; 0.83) CTC per ml, respectively (Figure 2A). Thus, significant differences were observed only when the CTCs co-expressed αVβ5 integrin with any other.

Analysis of β3, β4, and αVβ5 co-expression on CTCs did not reveal significant differences in patients with different localization of metastasis. Therefore, we evaluated the mono-expression of β3, β4, and αVβ5 integrins on CTCs in relation to the localization of distant metastases (Figure 2B).

Patients with liver metastases demonstrated an increased number of β4+ and αVβ5+ CTCs compared to patients without metastasis (*p* = 0.0242 and *p* = 0.032, respectively). The median level of CTC number with β4-positive expression in patients with lung and liver metastasis was 2.075 (0; 4.150) cells/mL and 4.565 (1.245; 42.745) cells/mL. The median level of CTC number with αVβ5-positive expression in patients with lung and liver metastasis was 2.905 (0.830; 4.980) cells/mL and 11.620 (3.943; 47.518) cells/mL. In patients without metastasis median values of β4+ and αVβ5+ CTCs were 1.66 (0; 4.150) cells/mL and 3320 (0.140; 10.375) cells/mL, respectively. 

We evaluated the intrapersonal heterogeneity of β3, β4, and αVβ5 integrins co-expression on CTCs of each patient (Figure 2C). The diversity of integrins phenotypes detected in CTCs varied from two to seven out of eight possible in patients with metastasis in distant organs. Patients without metastasis in the follow-up period demonstrates similar patterns and the diversity of phenotypes varied from one to seven (Figure 2C). 

### 2.3. Expression of β4 Integrin in Primary Tumor of Breast Cancer Patients

We studied the frequency of occurrence of integrins expression in the primary tumor tissue of breast cancer patients depending on the localization of hematogenous metastases. Integrin β4-positive cells were found in the tissue of all patients with hematogenous metastases, and in 80% of patients without metastases. 

**Figure 3:**
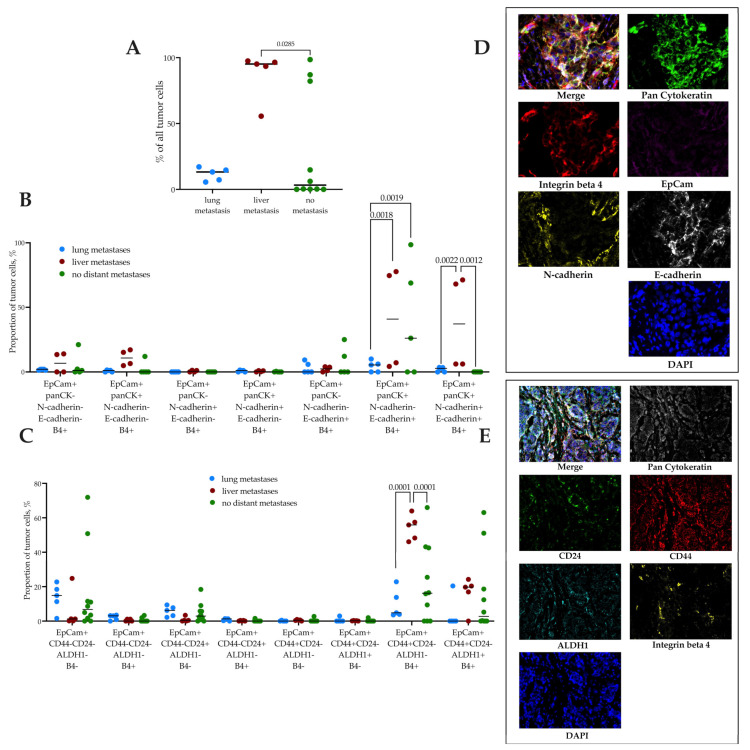
(**A**) Proportion of tumor cells expressed β4 integrin in breast cancer patients with distant metastases in different organs. (**B**) Proportion of integrin β4+ tumor cells with EMT features in breast cancer patients with distant metastases in different organs. (**C**) Proportion of integrin β4+ tumor cells with stemness features in breast cancer patients with distant metastases in different organs. (**D**,**E**) EMT (E-cadherin and N-cadherin) and stem (CD44+CD24- and ALDH1) markers in Integrin beta 4-positive tumor cells in breast cancer patients (Multiplex IHC, (**A**)—magnification 630×, (**B**)—magnification 400×). Samples were tested for statistical outliers, then one-way ANOVA was used to compare the means of independent groups.

The proportion of β4-positive cells was higher in patients with liver metastases (95.19 (74.54; 97.0)%) compared to patients without metastases (3.22 (0.12; 83.39)%, *p* = 0.0285) (Figure 3A,D,E). In-depth analysis of β4+ tumor cells’ phenotypic characteristics taking into consideration stemness and EMT features revealed a significantly increased proportion of E-cadherin+ (Figure 3B) and CD44+CD24- cells (Figure 3C) in patients with liver metastases compared to patients with lung or without distant metastases.

Integrin β4+ tumor cells associated with liver metastasis were characterized by EpCam+panCK+N-cadherin+E-cadherin+ phenotype (37.1 (6.18; 70.45)%), and their number was higher compared to patients without metastases (0 (0; 0)%, *p* = 0.0012) and than patients with lung metastases (2.80 (0; 3.45)%, *p* = 0.0022). Another population of β4+ tumor cells with the EpCam+panCK+N-cadherin-E-cadherin+ phenotype prevailed in patients with liver metastases (40.87 (5.01; 76.92), *p* = 0.0018) and in patients without metastases (26.17 (0; 88.71)%, *p* = 0.0019) compared with patients with lung metastases (5.42 (0–8.08)%) (Figure 3B).

## 3. Discussion

It has been experimentally shown that exosomal integrin β3 targets metastases to the brain, β4 to the lungs, and αvβ5 to the liver [2]. The authors convincingly demonstrated the decisive role of exosomes in premetastatic niche formation, showing that the tropism of exosomes, which can redirect tumor cell metastasis, is critical. The purpose of this study was to investigate the role of integrins in the targeting of metastases to specific organs using clinical samples obtained from breast cancer patients. The expression of exosomal integrins 3, 4, and αvβ5 was studied in inoperable patients with stage 4. In all patients with metastases, except one, exosomes with mono-expression of each integrin or exosomes with co-expression of two or three integrins were found. Therefore, in each case, it was possible to target tumor cells to more than one organ. For the implementation of multiple metastases, there was also the main condition—the presence of tumor cells with molecular characteristics of “seeds” [11]. This was evidenced by the presence of at least one localization of hematogenous metastases. Finally, the duration of the disease was sufficient to implement all the conditions necessary for the clinical manifestation of distant metastases with certain localization. 

Only four cases with isolated liver metastasis and three cases with isolated bone metastasis were detected in a group of studied patients. Most often metastases were found in two organs, and less often metastases occurred in three organs. The bones were involved in all cases of multiple metastasis.

Only exosomal β4 integrin mono-expression was associated with the presence of lung metastases, which is consistent with the available experimental data [2]. The presence of exosomes with the expression of integrin αvβ5 was not associated with liver metastases in our study. We were unable to evaluate the role of integrin β3 in brain metastasis due to the rarity of cases with this localization. It is known that β4 integrin is expressed in various tumors and plays a role in tumor invasion, proliferation, epithelial-mesenchymal transition, and angiogenesis. The extracellular domain of the integrin β4 binds to the basement membrane component laminin. In breast cancer, integrin β4 induces an invasive status, enhances the viability of tumor cells, and promotes angiogenesis [12]. It has been shown that the expression of α6β4 integrin on human breast cancer cells promotes lung metastasis due to interaction with human CLCA2 (hCLCA2) expressed on the endothelial cell of the inner surface of pulmonary arteries, arterioles, and venules [13].

The comparison of the group of inoperable metastatic patients with the group of patients without metastases revealed that the spectrum of exosomal integrins was not significantly different. In patients with metastases to parenchymal organs, the number of different phenotypes of exosomal integrins in each patient varied from two to seven phenotypes out of eight possible. In patients without metastases, diversity varied from three to eight phenotypes. Perhaps, in prospective observation, one can expect a similar association between the individual exosomal integrins expression and the localization of distant metastases.

It is noteworthy that the presence of cases with isolated metastasis is only in the liver and bones, as well as almost obligate bone involvement while metastases were combined. It appears that both liver and bone localization are least affected by selective targeting mediated by exosomal integrins. This may be why it is not possible to find specific exosomal integrins associated with bone metastasis. In the endothelium of the hepatic sinusoids there is no ligand named CLCA2 (hCLCA2), which is present in the lungs and could bind integrin β4. At the same time, a number of unique features characterize the endothelium of the hepatic sinusoids. Liver sinusoids are lined by a fenestrated endothelium that lacks a basement membrane. Fenestrated liver sinusoidal endothelial cells (LSECs) provide antioxidant activity, suppress inflammation in the liver, and promote regeneration. Defenestration of LSECs also occurs during chronic liver disease [14]. Defenestrated LSECs can cause inflammation in the liver, promote hematopoietic stem cell activation, and, most importantly, enhance tumor cell adhesion. Due to these properties, LSECs play an important role in carcinoma metastasis to the liver. Apparently, this explains the frequent localization of metastases in the liver [15]. 

As for frequent bone metastasis, the mechanism is probably different. In our study, isolated bone metastasis was characterized by minimal diversity and the number of exosomes with the studied integrins. The proportion of exosomes with integrin β4 was even lower than in other cases, while the proportion of exosomes without the studied integrins was higher than in the cases with metastases to other organs. It is known that, unlike other sites of metastasis, where the formation of premetastatic niches de novo is required, there are constitutive stem cell niches in the bone marrow that can act as premetastatic niches. Favorable conditions for the development of bone metastases are confirmed by the fact that in breast cancer, bone metastases are found in 70% of cases [16].

Analysis of the expression of integrins on CTCs showed that along with the mono-expression of one of the studied integrins, various co-expression variants are present. The frequency of distant metastasis, regardless of location, was associated with the expression of αVβ5 integrin with integrin β3 and/or β4. Integrin αVβ5 is known to be a pro-angiogenic factor [1,17]. In addition, integrin αvβ5 is believed to play a significant role in the invasion and metastasis of carcinomas [18]. Experimental studies have shown that the presence of exosomes with αvβ5 expression is associated with different locations of hematogenous metastases. There is evidence that the expression of αvβ5 integrin is associated with the development of liver metastases [2]. On the other hand, αvβ5 expression on melanoma cells promotes brain metastasis [18].

In our study, the presence of CTCs with integrin αvβ5 with different variants of co-expression was observed in patients with different localizations of metastases, however, the number of CTCs was significantly increased in the case of metastasis to the liver. Tumor cells in the primary site expressing integrin β4 were also associated with liver metastasis. Moreover, this relationship was found both for primary tumor cells and for CTCs. 

Thus, in our study, β4 integrin was associated with metastasis, wherein exosomal expression to the lungs, and expression on tumor cells (CTC and primary tumor) to the liver. Apparently, this could be explained by the fact that β4+ tumor cells associated with liver metastasis, also expressed EpCam, cytokeratins, E-cadherin, and N-cadherin, i.e., these cells could be in two states—without EMT manifestations or could have a hybrid EMT phenotype (in the case of co-expression of E-cadherin and N-cadherin). Moreover, the hybrid phenotype in this case could also arise during the mesenchymal-epithelial transition. There is evidence that it is cells with EMT hybrid phenotypes that express epithelial traits that initiate carcinoma metastasis [19]. In addition, β4+ tumor cells metastasizing to the liver had signs of stemness (EpCam+CD44+CD24-ALDH1-β4+).

Exosomes are produced in the cells in a non-random way, such as microvesicles, and many proteins are involved in their formation, providing selective packaging of the contents. Therefore, the question arises whether the integrin profile of exosomes repeats the profile of the tumor producer cell. In our study, the integrin profile of exosomes and tumor cells (CTCs) was characterized by a combination of mono- and co-expressions of the studied integrins but had frequency and quantitative differences. This agrees with the data that the expression of exosomal integrins does not necessarily reflect the expression of cellular integrins. This could be explained by the selective expression of integrins on exosomes. In our study, we also tried to trace the role of not only exosomal integrins, but also integrins on potential sources of exosomes—tumor cells of the primary tumor. We hypothesized that the targeting mechanism could be universal and not limited to targeting only exosomes as initiators of premetastatic niches. The correlation analysis in cases where we had data on the expression of integrins simultaneously in exosomes, CTCs, and tumor cells, did not reveal any no correlations. Thus, the relationship of exosomal integrins with metastasis, on the one hand, and integrins of the primary tumor and CTC, on the other hand, was different. Apparently, this is associated with different mechanisms of participation in the formation of metastases of exosomal and cellular integrins. Targeted by integrins to certain loci, exosomes can act as transporters of various bioactive molecules. They can carry phlogogenic agents such as pro-caspase-1, IL-1, 6, 18, components of inflammosomes and, thus, initiate the formation of premetastatic niches. While integrins on tumor cells are probably important for the selective attachment of CTCs to the endothelium.

Our study has a number of limitations. Exosomal integrins and integrins expressed by primary tumor in patients with M1 were not possible to compare because most of the patients did not undergo surgical treatment. There were no cases with brain metastases among the studied cases, therefore we cannot evaluate the association of integrin β3 with metastasis to the brain. We were unable to evaluate the association of specific integrins in M0 patients due to a short follow-up period. 

## 4. Materials and Methods

### 4.1. Patients

The prospective study included 71 patients with invasive breast carcinoma of no special type (IC NST) T1-4N0-3M0-1, admitted for treatment to Cancer Research Institute, Tomsk National Research Medical Center. The study was approved by the Local Committee for Medical Ethics of the institute (17 June 2016, approval No. 8), and informed consent was obtained from all patients prior to analysis. Venous ethylenediaminetetraacetic acid (EDTA) blood samples (n = 38) to assess the expression of integrins β3, β4, αV in CTCs of breast cancer. Wherein, 28 out of 38 breast cancer patients were taken before surgery and neoadjuvant chemotherapy, and 10 metastatic breast cancer patients were pretreated. Plasma samples of breast cancer patients (n = 24) were used for exosome isolation for further aim to study exosomal expression of integrins β3, β4, αV. True archival FFPE breast cancer cases (n = 20), stored at room temperature, were utilized to investigate the expression of integrin β4 in primary breast tumor cells. Patients were treated according to ESMO Clinical Practice Guidelines [20].

### 4.2. Isolation of Exosomes from Plasma 

Exosomes were isolated from the plasma of breast cancer patients by sequential differential centrifugation, according to a commonly used protocol with some modifications [21]. The blood samples were centrifuged at 300× *g* and 3000× *g* to remove erythrocytes and cell debris. Then, the supernatant was filtered using a 0.45 μm filter (Millipore Corp., Bedford, MA, USA) and centrifuged at 110,000× *g* for 80 min using Optima XPN-100 Ultracentrifuge (Beckman Coulter, Brea, CA, USA) (all steps were performed at 4 °C). Exosomes were harvested from the pellet and resuspended in sterile PBS. 

The preparation for transmission electron microscopy (TEM) included sorption of the exosomes on a copper grid covered with a formvar film for 1 min and contrasting with a 2% solution of phosphotungstic acid or an aqueous solution of uranyl acetate for 10 s. TEM images were taken with a JEOL 1400 TEM at an acceleration voltage of 120 keV and a Gatan Orius Camera.

### 4.3. Blood Specimen Collection and Processing for CTCs Immunophenotyping

Blood samples were collected in EDTA pre-coated 9 mL tubes, then incubated at 37 °C; for 1.5 h. White blood cells were aspirated from a thin white layer between plasma and red blood cells after their sedimentation. Obtained cell concentrate washed in 2 mL Cell Wash buffer (BD Biosciences, San Jose, CA, USA) by centrifugation at 800× *g* for 15 min.

### 4.4. Flow Cytometry

#### CTCs Immunophenotyping

Surface markers (CD45, EpCAM (CD326), integrins β3, β4, and αVβ5) were stained in the first step, intracellular staining was performed during the second step. Samples were incubated at RT for 10 min with 5 μL of Fc Receptor Blocking Solution (Human TruStain FcX, Sony Biotechnology, San Jose, CA, USA). Next, monoclonal antibodies were added and incubated at RT for 20 min: APC-Cy7-anti-CD45 (clone HI30, IgG1, Sony Biotechnology, USA), BV 650-anti-EpCAM (clone 9C4, IgG2b, Sony Biotechnology, USA), BV 421-anti-β3 integrin (clone VI-PL2, BD Biosciences, USA), Alexa Fluor 488-anti-β4 integrin (clone 422325, R&D Systems, Minneapolis, MN, USA), BV Alexa Fluor 750-anti-αVβ5 integrin (clone P5H9, R&D Systems, USA). The unstained control and antibody quality control were performed. The appropriate isotype antibodies were added to the isotype control sample at the same concentration. After incubation, red blood cells were lysed by 250 μL OptiLyse C buffer (Beckman Coulter, Villepinte, France) at RT for 10 min in dark and washed in 1mL Cell Wash buffer (BD Biosciences, USA) at 800× *g* for 6 min.

For intracellular staining, cells were permeabilized by 250 μL BD Cytofix/Cytoperm (BD Biosciences, USA) at 4 °C for 30 min in the dark and washed twice in 1mL BD Perm/Wash buffer (BD Biosciences, USA) at 800× *g* for 6 min. Then, samples were diluted in 50 μL BD Perm/Wash buffer (BD Biosciences, USA) and incubated at 4 °C for 10 min in the dark with 5 μL of Fc Receptor Blocking Solution (Human TruStain FcX, Sony Biotechnology, USA). Next, monoclonal antibodies BV 650-anti-EpCAM (clone 9C4, IgG2b, Sony Biotechnology, USA) were added and incubated at 4 °C for 20 min. The appropriate isotype control antibodies at the same concentration were added to the control sample. After incubation, samples were washed in 1mL Cell Wash buffer (BD Biosciences, USA) at 800× *g* for 6 min. Then, samples were diluted in 100 μL Stain buffer (Sony Biotechnology, USA). Compensation beads (VersaComp Antibody Capture Bead kit, Beckman Coulter, USA) were used for compensation control. The immunofluorescence was analyzed on the Novocyte 3000 (ACEA Biosciences, San Diego, CA, USA). 

The gating strategy was as follows: using forward (FSC) and side scatter (SSC), debris were descriminated, and doublets were also discriminated by plotting FSC area vs. FSC height. Further analysis included only CD45-negative and EpCAM-positive cells. Then, we characterized CTCs by the expression of integrins β3, β4, and αVβ5. 

### 4.5. Exosomes Immunophenotyping 

Obtained exosomes were stained with PE-anti-CD81 (clone SA6, Sony Biotechnology, USA), BV 421-anti-β3 integrin (clone VI-PL2, BD Biosciences, USA), BV 650-anti-αVβ5 (clone ALULA, BD Biosciences, USA) antibodies and left for 20 min at RT. Next, 900 μL of sterile PBS was added to the samples before the acquisition on the CytoFLEX flow cytometer (Beckman Coulter, Brea, CA, USA). The cytometer was calibrated using a mixture of non-fluorescent Flow Cytometry Sub-micron Size Reference Kit (Invitrogen, Waltham, MA, USA) with sizes ranging from 0.02 µm to 2.0 µm. This calibration step enabled the determination of the sensitivity and resolution of the flow cytometer and the size of extracellular vesicles. All samples were acquired at a high flow rate and high dilution to avoid a coincidence or swarm detection. The analysis of the data was performed with CytExpert software (Beckman Coulter, Brea, CA, USA).

### 4.6. Multiplex Immunofluorescent Analysis

#### Primary Tumor Cells Immunophenotyping

For stem and EMT features evaluation, 4-μm tissue sections were stained according to a well-established protocol in a Bond-RXm immunostainer (Leica, Munich, Germany). The following panels of primary antibodies were used: (1) Anti-panCK (1:10, clone AE1/AE3, Roche, Indianapolis, IN, USA), Anti-EpCam (1:500, clone E144, Abcam, Cambridge, UK), Anti-E-cadherin (1:10, clone EP700Y, CellMarque, USA), Anti-N-Cadherin (1:500, clone EPR1791-4, Abcam, UK), Anti-β4 integrin (1:400, clone JM11-06, Thermo, Waltham, MA, USA); (2) Anti-panCK (1:10, clone AE1/AE3, Roche, Indianapolis, IN, USA), Anti-CD24 (1:100, clone SN3b, Thermo), Anti-CD44 (1:100, clone 156-3C11, Thermo, USA), Anti-ALDH1 (1:500, polyclonal, Abcam, UK), Anti-β4 integrin (1:400, clone JM11-06, Thermo, USA). A 7-color Opal Kit (NEL797B001KT; PerkinElmer, Waltham, MA, USA) containing Opal 520, Opal 540, Opal 570, Opal 620, Opal 650, and Opal 690 fluorophores was used. The obtained slides were embedded in Mounting Medium with DAPI (VECTOR Laboratories Inc., Newark, CA, USA). The slides were scanned using Vectra 3.0 system (PerkinElmer, USA). Images were processed using inForm 2.2.1 software (PerkinElmer, USA). The frequency and the number of tumor cells in different populations as a percent of all tumor cells were assessed.

### 4.7. Statistical Analysis

The data were analyzed using GraphPad Prism 9 (GraphPad Software, San Diego, CA, USA). The one-way ANOVA was used for multiple comparisons between independent groups. Fisher’s exact test was applied to assess differences in the frequency of integrin expression in breast cancer patients with different localization of distant metastasis. *p* < 0.05 was considered statistically significant. 

## 5. Conclusions

On the one hand, the results of the study confirm the role of integrins expressed on exosomes and tumor cells (CTCs and primary tumor) in organotropic metastasis. This concerns the association of exosomal expression of integrin β4 with lung metastases, and cellular expression with liver metastases. On the other hand, the presence of exosomes and/or CTCs with different integrins, which could simultaneously initiate the development of metastases in other organs, in patients with isolated metastases, did not lead to this. The presence of the other site-specific integrins on exosomes and/or CTCs did not result in metastases development, as one can assume. For example, almost all patients had an exosomal and cellular expression of integrin β3, however, brain metastasis did not occur. One can explain such phenomenon by polycausality of metastasis in clinical conditions, wherein integrin-mediated targeting could take place, but other limitation factors could play a more significant role. The results suggest that metastasis to the liver and bone is least related to specific targeting by integrins, but further clarification of the role of co-expression of integrins is needed.

## Figures and Tables

**Table 1:** Clinicopathological characteristics of patients.

Parameter		Frequency, % (n)	
Age		First cohort	Second cohort
	<35	6.25% (3/48)	5.56% (1/18)
	35–50	37.50% (18/48)	33.33% (6/18)
	>50	56.25% (27/48)	61.11% (11/18)
Tumor size (T)	1	31.25% (15/48)	5.56% (1/18)
	2	56.25% (27/48)	38.88% (7/18)
	3	2.08% (1/48)	5.56% (1/18)
	4	10.42% (5/48)	50.00% (9/18)
Molecular subtype	Luminal A	29.17% (14/48)	5.56% (1/18)
	Luminal B	58.33% (28/48)	83.33% (15/18)
	Triple negative	8.33% (4/48)	0.00% (0/18)
	HER2 positive	4.17% (2/48)	11.11% (2/18)
Estrogen receptor α	positive	81.25% (39/48)	83.33% (15/18)
	negative	18.75% (9/48)	16.67% (3/18)
Progesterone receptor	positive	66.67% (32/48)	66.67% (12/18)
	negative	33.33% (15/48)	33.33% (6/18)
HER2	positive	28.79% (10/48)	50.00% (9/18)
	negative	71.21% (38/48)	50.00% (9/18)
Ki67 expression	<20%	20.83% (16/48)	44.44% (8/18)
	>20%	79.17% (32/48)	55.56% (10/18)
Metastasis in follow-up period	no metastasis	75.00% (36/48)	100.00% (18/18)
	lymph node metastasis	6.25% (3/48)	0.00% (0/18)
	distant metastasis	18.75% (9/48)	0.00% (0/18)

**Table 2:** Frequency of occurrence of β3, β4, and αVβ5 integrins co-expression on exosomes of breast cancer patients.

№	Phenotype	Frequency	Significance Level *
1	β3+β4+αvβ5+	79.2% (19/24)	*p*_1-2_ = 0.0687; *p*_1-3_ = 0.0077
2	β3+β4+αvβ5-	50% (12/24)	
3	β3+β4-αvβ5+	37.5% (9/24)	
4	β3+β4-αvβ5-	54.2% (13/24)	
5	β3-β4+αvβ5+	50% (12/24)	
6	β3-β4+αvβ5-	95.8% (23/24)	*p*_6-2_ = 0.0007; *p*_6-3_ = 0.0001; *p*_6-4_ = 0.0018; *p*_6-5_ = 0.0007; *p*_7-6_ = 0.0044
7	β3-β4-αvβ5+	58.3% (14/24)	
8	β3-β4-αvβ5-	95.8% (23/24)	*p*_8-2_ = 0.0007; *p*_8-3_ = 0.0001; *p*_8-4_ = 0.0018; *p*_8-5_ = 0.0007; *p*_8-7_ = 0.0044

* Note: two-tailed Fisher’s exact test.

**Table 3:** Frequency of occurrence of β3, β4, and αVβ5 integrins co-expression on CTCs of breast cancer patients.

№	Phenotype	Frequency	Significance Level *
1	β3+β4+αvβ5+	18.4% (7/38)	
2	β3+β4+αvβ5-	36.8% (14/38)	
3	β3+β4-αvβ5+	50% (19/38)	*p*_3-1_ = 0.0072
4	β3+β4-αvβ5-	68.4% (26/38)	*p*_4-1_ = 0.0001; *p*_4-2_ = 0.0110
5	β3-β4+αvβ5+	44.7% (17/38)	*p*_5-1_ = 0.0253; *p*_5-4_ = 0.0634
6	β3-β4+αvβ5-	52.6% (20/38)	*p*_6-1_ = 0.0036
7	β3-β4-αvβ5+	60.5% (23/38)	*p*_7-1_ = 0.0003
8	β3-β4-αvβ5-	92.1% (35/38)	*p*_8-1_ < 0.0001; *p*_8-2_ < 0.0001; *p*_8-3_ = 0.0001; *p*_8-4_ = 0.0190; *p*_8-5_ = 0.0001; *p*_8-6_ = 0.0002; *p*_8-7_ = 0.0024

* Note: two-tailed Fisher’s exact test.

## Data Availability

The data presented in this study are available on request from the corresponding author. The data are not publicly available due to data being prepared for intellectual property approval.
